# Post-Acute COVID-19 Neurological Syndrome: A New Medical Challenge

**DOI:** 10.3390/jcm10091947

**Published:** 2021-05-01

**Authors:** Domenico Nuzzo, Sonya Vasto, Luca Scalisi, Salvatore Cottone, Gaetano Cambula, Manfredi Rizzo, Daniela Giacomazza, Pasquale Picone

**Affiliations:** 1 Consiglio Nazionale delle Ricerche (CNR), Istituto per la Ricerca e l’Innovazione Biomedica (IRIB), 90146 Palermo, Italy; 2 Dipartmento of Scienze Biologiche, Chimiche, Farmaceutiche e Tecnologiche (STEBICEF), University of Palermo, 90128 Palermo, Italy; sonya.vasto@unipa.it; 3 Azienda Sanitaria Provinciale Di Trapani (ASP 9 TP), 91100 Trapani, Italy; luca.scalisi@villasarina.com; 4 Azienda Ospedaliera di Rilievo Nazionale e di Alta Specializzazione “Civico Di Cristina e Benfratelli”, 90127 Palermo, Italy; salvatore.cottone1@arnascivico.it; 5 Unità Operativa Complessa Radiologia P.O.S. Antonio Abate-Azienda Sanitaria Provinciale di Trapani, 91100 Trapani, Italy; gaetanocambula@libero.it; 6 Department of Health Promotion, Mother and Child Care, Internal Medicine and Medical Specialties, University of Palermo, 90128 Palermo, Italy; manfredi.rizzo@unipa.it; 7 Istituto di Biofisica (IBF), Consiglio Nazionale delle Ricerche, 90146 Palermo, Italy; daniela.giacomazza@cnr.it

**Keywords:** COVID-19, SARS-CoV-2, neurology, brain damage, post-scute COVID-19 neurological syndrome

## Abstract

In December 2019, in Wuhan (China), a highly pathogenic coronavirus, named SARS-CoV-2, dramatically emerged. This new virus, which causes severe pneumonia, is rapidly spreading around the world, hence it provoked the COVID-19 pandemic. This emergency launched by SARS-CoV-2 also had, and still has, devastating socio-economic aspects. Assessing the impact of COVID-19 on vulnerable groups of people is crucial for the adaptation of governments’ responses. Growing scientific evidence suggests that it is essential to keep the attention on people after acute SARS-CoV-2 infection; indeed, some clinical manifestations are frequently present even after recovery. There is consensus on the need to define which symptoms persist after the infection and which disabilities may arise after COVID-19. Recent reviews, case reports, and original contributions suggest that various organs may be affected, and neurological symptoms are present in about one third of patients with COVID-19. Neurological complications after severe COVID-19 infection might include delirium, brain inflammation, stroke, and nerve damage. In the recent pandemic, neurologists and neurobiologists have a chance to study key features of infection neurology. Furthermore, the psychological impact of the pandemic should not be underestimated, although there is currently no definition for this condition.

## 1. Introduction

In December 2019, in Wuhan, one of the most populous cities in Central China, the current coronavirus disease 2019 (COVID-19) pandemic triggered by severe acute respiratory syndrome coronavirus type 2 (SARS-CoV-2) was identified. In January 2020, the World Health Organization (WHO) declared a public health emergency of international alarm. In fewer than 10 months, SARS-CoV-2 spread worldwide, killing over 2,300,000 people with more than 149,000,000 confirmed infected cases as of today (https://covid19.who.int) (29 April 2021). The global COVID-19 pandemic crisis was never verified before, creating a massive disruption to lives and livelihoods, as well as to social and economic systems around the world. This virus is highly contagious and spreads steeply everywhere in the world. Young people act as asymptomatic carriers, but older and frail people are more likely to have the most severe form of the disease and higher mortality. The severe form of COVID-19 infection can have profound implications in the short- and in the medium-term, but we postulate there are implications in the long-term, too. Before identification of the present pandemic disease, six other human infectious coronaviruses were identified, and four of them (HCoV-229E, -OC43, -NL63, and -HKU1) still have a high incidence worldwide and are responsible for 15%–30% of cases of upper respiratory tract infections [1]. The last two gave origin to serious epidemic respiratory diseases; SARS was caused by SARS-CoV in 2002–2003, and Middle East Respiratory Syndrome (MERS) by MERS-CoV, which was firstly identified in Saudi Arabia in 2012 [2,3]. Rare diseases of the central nervous system (CNS) and the peripheral nervous system (PNS) are related to them [4]. For SARS-CoV-2, the incubation period varies from 4 to 11 days [5]. Common symptoms include loss of smell and taste, fever, cough, sore throat, and feeling of malaise. Some gastrointestinal symptoms are also frequent, such as diarrhea, nausea, and anorexia [6]. Risk factors of complications due to the infection from SARS-CoV-2 include an age above 65 years, chronic lung disease, cardiovascular diseases, hypertension, diabetes, and obesity [7,8,9,10,11].

The manifestation of severe SARS-CoV-2 infection is characterized by sepsis and acute respiratory failure. About 20% of the hospitalized patients positive for SARS-CoV-2 infection develop a strong sequelae of serious symptoms requiring intensive care and oxygen supplementation [12]. Hui and coworkers reported characteristic signs of shock in critical COVID-19 patients, including cold extremities, weak peripheral pulses, and severe metabolic acidosis, indicating a possible dysfunction at the microcirculation level [13]. In a recent meta-analysis, neurological damage was evaluated to vary from patient to patient, from the ones with nonspecific symptoms to those with specific symptoms. Some severe symptoms can also become visible in the later stage of the disease [14]. Furthermore, it was recently remarked that, in addition to the symptoms involving the respiratory system, 36.4% of hospitalized patients with severe SARS-CoV-2 have a higher probability to experience neurological symptoms, including headache, altered consciousness, and paresthesia compared to those who have mild/moderate disease [15].

In France, 84% of the patients with COVID-19 showed neurological complications, such as encephalopathy (69%) and signs of the corticospinal tract (67%) [16].

We previously reported that SARS-CoV-2 infection may cause brain damage through both a direct way (the virus directly reaches the brain) and an indirect effect (high systemic inflammation and oxidative stress) [17]. In May 2020, Moriguchi’s group first reported a case of aseptic encephalitis after SARS-CoV-2 infection with the discovery of the SARS-CoV-2 RNA in the cerebrospinal fluid of the patient. This case report was described in a 24-year-old man with seizures followed by unconsciousness [18]. Several published articles suggest that some COVID-19 patients may develop post-acute neurological, cardiovascular, and renal alterations. In a recent study presented by Carfì and collaborators, the authors found out that 87.4% of patients recovered from SARS-CoV-2 had persistence of at least one symptom [19].

A recent study of follow-up of adults with non-critical COVID-19 showed that, in the medium-term, two-thirds of patients still reported symptoms at 30 and 60 days, and one third were still sick or in a worse clinical condition at 60 days [20]. Furthermore, the cohort of patients that still presented symptoms after 60 days belonged to the 40–70 age range [20].

Therefore, the COVID-19 pandemic requires a careful medical treatment even after its end. It should be noted that the study presented some limitations because it was a single-center study, and only non-critical COVID-19 patients were included.

The paper published by Garrigues and coworkers showed that most patients needing hospitalization for COVID-19 still presented persistent symptoms such as fatigue and dyspnea, even 110 days after discharge. After discharge, the most usual reported symptoms were fatigue (55%), dyspnea (42%), memory loss (34%), sleep disorders such as insomnia, (30.8%), and concentration difficulty (28%). These findings emphasize the need for long-term follow-up of those patients and rehabilitation programs [21,22]. One of the weaknesses of the study is that it was based on the patient’s self-assessment, and for this reason, they could have undergone incomplete recall or recall bias.

From what was said above, it appears that post-acute COVID-19 syndrome is a multisystem disease that can occur even after a relatively mild infection, although these conditions seem to affect mainly COVID-19 patients with more severe forms of the disease. Currently, there is no clear definition of these clinical alterations.

**Figure 1:**
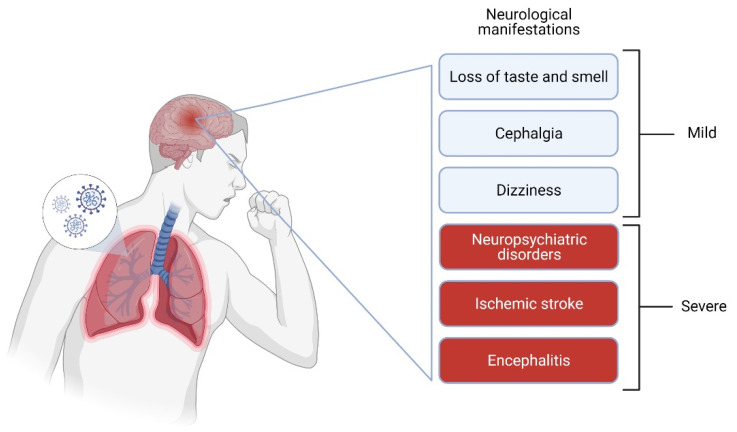
Neurological disorders during and after COVID-19. Created with BioRender.com.

In this review, we discuss some recognized neurological signs of SARS-CoV-2 infection. We believe that it is necessary to highlight the presence of neurological signs during and after SARS-CoV-2 infection, although, thus far, little is known to treat the disease and prevent a worsening of patients’ prognosis. Neurological manifestations of COVID-19 (Figure 1) vary from mild (e.g., loss of taste and smell, cephalgia, dizziness) to severe (e.g., ischemic stroke, encephalitis).

## 2. Neurobiology of SARS-CoV-2 Infection

Several studies recently showed that symptoms affecting the nervous system, such as sensory disturbances, headache, nausea, and vomiting, may occur in patients with SARS-CoV-2 infection and persist after infection [17]. Here, we discuss the different neurobiological processes and mechanisms linking SARS-CoV-2 to neuronal dysfunction.

## 3. Potential Mechanisms for the Penetration of SARS-Cov2 in CNS

The potential pathways of SARS-CoV-2 neuro-invasion from the periphery to the brain are many. All these routes are determined by the angiotensin-converting enzyme-2 receptor (ACE2), used by SARS-CoV-2 to bind and penetrate human cells [23]. This receptor is broadly distributed in lungs, heart, liver, kidney, intestine, and oral and nasal mucosa.

### 3.1. Direct Route

The existence of the direct route is hypothesized with the aid of coronavirus-infected animal experiments [24]. Possibly, after the droplets containing SARS-CoV-2 reach the nasal cavity, most viruses head to the lung, while others adhere to the mucosa of the nasal cavity from which they directly attack the olfactory sensory neurons in the olfactory epithelium; from here, they can be transferred into the CNS through the olfactory nerve. In addition, the abundant capillary blood vessels and lymphatics existent in the nasal mucosa provide chances for virus invasion of the bloodstream, from which they reach the brain. Moreover, SARS-CoV-2, adhering to the nasal mucosa or accessing the eye conjunctiva, may also reach the trigeminal nerve and then the brain through the route used for brain drug delivery [25]. The virus may also infect the sensory neurons in the taste buds, reach the nucleus of the solitary tract (VII, IX, and X) or the trigeminal nerve (V), and enter the CNS through neuronal retrograde transport [23].

Recently, Song and collaborators suggested the direct neuro-invasion of SARS-CoV-2 in the central nervous system in human and mouse brains. They showed that the brain is a site for high SARS-CoV-2 replication, with metabolic changes in neurons (neuronal death) in human brain organoids. Neuronal infection can be excluded by blocking ACE2, with antibodies, or by administering cerebrospinal fluid from a COVID-19 patient. Furthermore, using mice overexpressing human ACE2, the same authors demonstrated SARS-CoV-2 neuro-invasion in vivo. Finally, using COVID-19 brain autopsy of deceased patients, the authors detected SARS-CoV-2 in cortical neurons [26].

### 3.2. Indirect Route via Bloodstream

Viruses, once entered in the lungs, flow into the bloodstream through ACE2 receptor, present in the epithelium of the respiratory system. Moreover, they can also reach the gastrointestinal tract and then reach the brain through the vasculature system [23]. Once it has entered the bloodstream, the virus can infect the endothelial cells of the blood–brain barrier (BBB) or the blood–cerebrospinal fluid barrier (BCSFB), and then disseminate toward the CNS via ACE2 receptors. Besides, SARS-CoV-2′s cytokine storm in severely affected patients damages the BBB, resulting in infiltration of inflammatory factors and other blood contents, including viral particles, in the CNS [26]. Viruses in the blood can also directly enter the fourth ventricle through a damaged BCSFB.

## 4. SARS-CoV-2: Cellular Mechanism

Spike (S) proteins are essential for the virus to enter into the host cells. These proteins bind to the cellular ACE2 receptor, which is also present in neurons [27,28]. Protein S is the surface glycoprotein of the virus responsible for its crown shape. This protein is composed by two subunits, S1 and S2 [24]. The S1 subunit consists of the N-terminal domain (NTD) and the C-terminal domain (CTD). The receptor-binding domain (RBD) in the CTD is responsible for binding to the host cell. The S2 subunit allows the fusion with membranes. Full-length protein S, RBD domain, S1 subunit, and NTD are used as antigens to develop SARS-CoV-2 vaccines, including adenoviral, RNA-based, DNA-based, and protein subunit vaccines [29].

Furthermore, after intravenous injection, radioiodinated S1 (I-S1) protein was shown to cross the BBB in mice, be absorbed by brain regions, and enter the parenchymal brain space. Intranasally administered I-S1 penetrated the brain, but the levels were approximately ten times lower than those observed after intravenous administration. I-S1 intersected the BBB by transcytosis, and ACE2 was involved in the brain uptake [30].

**Figure 2:**
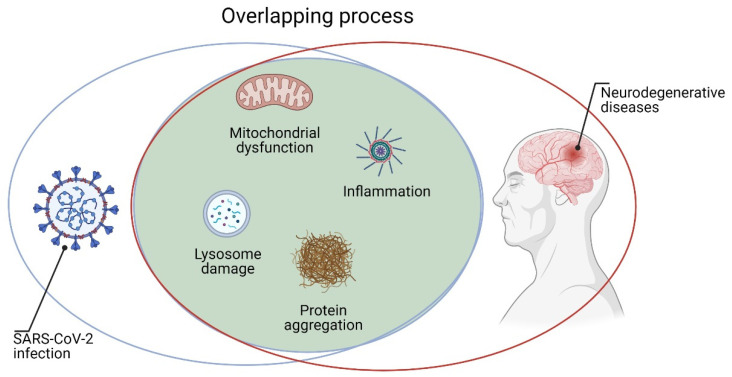
Similar alterations in SARS-CoV-2 infection and neurodegenerative processes. Created with BioRender.com.

The penetration of SARS-CoV-2 into neurons can alter their cellular processes for energy production and protein folding [29]. Inside the cell, the virus can cause mitochondrial dysfunction and lysosome damage by inducing increased reactive oxygen species (ROS), protein misfolding/aggregation, and, ultimately, cell death [26]. It is important to underline that all these processes are involved in the pathogenesis of various neurodegenerative diseases such as Alzheimer’s disease (AD), Parkinson’s disease (PD), and amyotrophic lateral sclerosis (ALS) [31,32,33]. Moreover, binding of ACE2 to the SARS-CoV-2 spike protein can reduce the conversion of angiotensin 2 (AT2) to AT [27]. Higher levels of AT2 are associated with pro-inflammatory markers and vascular injury to brain cells and other organs, all processes involved in neurodegeneration [27]. Furthermore, high levels of inflammation (cytokine storm) and BBB lesions in the brain are very likely to have long-term consequences on neurodegeneration. Indeed, there is indication that brain inflammation may contribute to the pathology of neurodegenerative diseases, including AD, PD, and ALS [34]. Coronaviruses such as SARS-CoV-2 could remain inside neurons without inducing toxic effects [35]. Therefore, in patients with acute SARS-CoV-2 infection, the virus could theoretically cause brain degeneration decades later [31]. Consequently, it would be useful to follow patients who were affected by COVID-19, allowing one to establish the real relationship between viral infection and neurodegenerative disorders (Figure 2). Finally, a better understanding of cellular and molecular mechanisms through which CoVs induced neuronal damage could help in performing new therapeutic strategies.

## 5. Neuroimmunology of COVID-19 Infection

Viruses stimulate the innate immune cells that recognize the molecular patterns associated with the pathogens. Decrease of the efficiency of the innate immune responses in eliminating the virus leads to the activation of adaptive immune system. The immune cells secrete cytokines and/or chemokines such as interleukin-6 (IL-6), interferon-γ (IFN-γ), interferon-γ-inducible protein-10 (IP-10), and monocyte chemoattractant protein-1 (MCP)-1. These molecules promote the influx of monocytes/macrophages and neutrophils to the site of infection [36]. Generally, this response is able to eliminate the virus, but occasionally the immune system is dysregulated, which leads to the alteration of the immune homeostasis [37]. SARS-CoV-2 infection can cause an intense inflammatory response, cytokine storm, which promotes lung pathogenesis and respiratory failure. This condition is one of the reasons for the higher mortality rate in fragile populations. As previously described, during COVID-19, the immune system is anomalously involved, and this may exacerbate autoimmunity in genetically predisposed people. Abnormal activation of immunological pathways could trigger mechanisms that alter the molecular recognition between viral epitopes and autoepitopes. Exacerbated immune-mediated manifestations are described in COVID-19 patients and represent the first sign of infection in fragile individuals. In addition, a robust adaptive immune response is essential for a conclusive viral clear and avoiding a re-infection [37].

During severe infection, the SARS-CoV-2 is able to infect macrophages, microglia, and astrocytes in the brain, which are particularly important. This neurotropic virus can stimulate glial cells and induce a pro-inflammatory status [38]. The most important cytokine during COVID-19 is interleukin 6 (IL-6), which is positively correlated with the severity of COVID-2019 symptoms. High levels of proinflammatory cytokines can cause confusion and alteration of consciousness [39]. A high level of inflammation may be associated with thrombophilia and increase the risk of stroke and other thrombotic events. The complementary activation may additionally lead to thrombotic microvascular injury in patients with severe COVID-19 [40]. Mohamud and coworkers reported five events of acute ischemic stroke simultaneous or 14 day delayed in patients with SARS-CoV-2 infection [41]. The mechanism of this event can be explained considering that the virus induces a high infiltration of macrophages, neutrophils, and T cells in the atheromatous plaques present in the blood vessel, exposing the patient to a thrombotic event. This can occur at the level of the carotid arteries [42]. The inflammation caused by SARS-CoV-2 infection can cause thinning of their fibrous caps, invasion of lipids, and expansion of the lipid layer [41].

High levels of cytokine and chemokine liberation may additionally lead to brain injury through microglial activation. In fact, in a case report, marked microglial nodules and neuronophagia were found in the brain tissue of a patient with COVID-19 [43].

## 6. Clinic Manifestation of Neuropathology of COVID-19 Infection

Neurological manifestations of COVID-19 infection are increasing to date. Ellul and collaborators elucidated the clinical features associated with COVID-19 infection in either central or peripheral nervous systems. Usually, neuropathological manifestations appear at the same time as the viral respiratory infection or some days after. Unfortunately, early reports did not have sufficient details to detect the presence of obvious neurological manifestations [4]. As the pandemic progresses, reports of neurological manifestations are increasing. To date, a multicenter study reported neurological manifestation as follows: three patients with encephalitis, 69 patients with other encephalopathies, and three patients with encephalomyelitis. Information relative to these patients is limited, but three died and, in three patients, neurological symptoms persisted long enough to require rehabilitation. Regarding peripheral nervous system manifestations, the study reports: seven patients with Guillain-Barrè disease diagnosed at the beginning of the viral infection and five patients with other neuropathies including Rhabdomiolysis. Among these patients, three needed rehabilitation, another three reported a longer time in intensive unit, and the rest reported minor or no information [44]. Furthermore, in 34 patients with cerebrovascular disease (ischaemic stroke and attacks), 20 died and six needed rehabilitation [4]. Loss of smell (anosmia) and taste (ageusia) emerged as common symptoms of COVID-19 and were present in 357 patients out of 417 in a European study [45] and in 68 out of 259 patients in a French retrospective observation [46]. There was no information on patients with abnormal smell and taste in persistent neurological symptoms. In the study of Varatharaj and colleagues at the beginning of 2020, among 125 patients recruited, the following percentage reported neurological problems: 13% with encephalopathy, 18% with a neuropsychiatric diagnosis (psychosis, neurocognitive syndrome, and an affective disorder), and 62% patients with cerebrovascular events [47]. Table 1 shows the neurological symptoms, the percentage of affected people, and the time of symptom onset.

In a pediatric multicenter neurological study, Lindan and collaborators found that the entire cohort studied did well with COVID-19 infection, whereas all children with hospital-acquired co-infections died, and two children were severely impaired and needed rehabilitation [48].

Although, the percentage of patients needing rehabilitation due to neurological associated COVID-19 manifestation is low, this is certainly due to the lack of follow up description after the hospital period of infection. At the beginning of the pandemic period, several observational studies lacked neurological description, implying that neurological manifestations were not evident. Today, it is quite clear that neurological traits associated with COVID-19 infection can be considered a sequel of the viral infection. Rogers and collaborators in a systematic review and meta-analysis showed how anxiety, fatigue, depression and post-traumatic stress were present in people surviving the COVID-19 infection as long-term manifestation. The etiology of the outcomes of infection with COVID-19 is likely to be multifactorial and might include the direct effects of viral infection (including encephalitis), cerebrovascular disease, physiological impairment (such as hypoxia), immunological activation, social isolation, and psychological impact. Many survivors complain about memory, attention, concentration, or mental impairments even after one year [49]. Eventually, it is quite possible that the psychiatric outcomes could be unrelated to the COVID-19 infection and may rather be a consequence of literature selection bias. At the same time, literature reporting post-illness studies did not agree on follow-up times and the studies cannot be compared. Therefore, thus far, there are too few studies for drawing conclusions but there is enough information to hypothesize that, in select populations, COVID-19 infection might drive neurological and psychiatric drawbacks.

## 7. Rehabilitation of Patients after COVID-19 Infection

There are not specific brain treatments for COVID-19 neurological effects. However, the German Society of Neurology published the first neurological manifestation guideline for the care of patients with neurological disease with and without SARS-CoV-2 infection. Moreover, an abbreviated version of the guideline was published [50].

Health care systems are currently focused on managing COVID-19 and its consequences on human health. The severity of the illness is related to advanced age, respiratory and cardiovascular diseases, hypertension, diabetes, and patients with immunodeficiency [51]. The initial personal visit should target the establishment of a patient’s baseline after SARS-CoV-2 infection. This procedure would require a deep investigation of present and past medical, social, and family history, physical examination, and blood testing. Once the COVID-19 post infection baseline is established, a follow-up evaluation during a visit should aim for a better understanding of the natural course of the disease and identify earlier new abnormalities. During the first 12 months of follow-up, clinical visit could be accompanied by some additional evaluations, such as SARS-CoV-2 infectivity testing lung analyses, 6 minute walk tests, quality of life monitoring, fatigue, and general blood tests.

It is now evident that this infection does not only involve the respiratory system but has consequences that affect cardiovascular, neurological, and musculoskeletal systems as well as other organs [52,53,54,55]. Furthermore, patients who stay longer in the intensive care unit may also develop a post-intensive care syndrome with anxiety, depression, or other neurological disorders. In addition to paying attention to the respiratory syndrome, it is necessary to evaluate and treat the after-effects derived from a condition with prolonged bed rest and invasive mechanical ventilation. The rehabilitation of patients after COVID-19 infection cannot be separated from medical assistance focused to the treatment of respiratory, neurological, and post-infectious pathologies [56]. Therefore, the rehabilitation program requires a multidisciplinary approach that responds in a highly specific way to the needs of the individual. The rehabilitation treatment concerns two fundamental aspects: (a) respiratory function [57] and (b) physical re-education of peripheral muscle function and increase of exercise capacity [58]. The first rehabilitation event is to re-educate the patient to postural passages in bed from supine decubitus to lateral decubitus (rolling activity). The positioning of the posture should be chosen based on thoracic/CT ultrasound, auscultation, and SpO2 [58]. The pulmonary rehabilitation program, physical exercise and aerobic training, was reconfirmed as a fundamental moment in the management of COVID-19 patients, together with neuromotor rehabilitation techniques and rehabilitation activities for peripheral muscles, strength, static and dynamic balance, and walking to improve autonomy and quality of life and the reintegration of the individual into society.

## 8. Conclusions

Despite the fact that neurological manifestations of COVID-19 are not yet fully investigated, some patients after a severe infection, frail people in particular, have CNS involvement for a long time [59]. A precise and targeted study of neurological symptoms can clarify the role played by the virus in causing prolonged neurological manifestations. It is essential to better define the characteristics of post-acute COVID-19 neurological syndrome to identify methods of intervention.

## Figures and Tables

**Table 1:** Neurological symptoms of COVID-19.

Neurological Symptoms (Percentage of Affected Persons)	Symptoms	References
Memory loss (34%), sleep disorders (30.8%), difficulty with concentration (28%).	100 days after hospitalization	[21]
Dizziness (16.8%), headache (13.1%), taste impairment (5.6%), and smell impairment (5.1%).	Hospitalized patients	[15]
Encephalopathy (13%), neuropsychiatric diagnosis (psychosis, neurocognitive syndrome, and an affective disorder) (18%), and cerebrovascular events (62%).	Reported over a 3 week period	[47]
Neurological complications (84%), including encephalopathy (69%) and corticospinal tract signs (67%).	Hospitalized patients	[16]
Encephalitis (case report)	Hospital	[18]

## References

[1] Desforges M., Le Coupanec A., Dubeau P., Bourgouin A., Lajoie L., Dubé M., Talbot P.J. (2019). Human coronaviruses and other respiratory viruses: Underestimated opportunistic pathogens of the central nervous system?. Viruses.

[2] Ksiazek T.G., Erdman D., Goldsmith C.S., Zaki S.R., Peret T., Emery S., Tong S., Urbani C., Comer J.A., Lim W. (2003). A novel coronavirus associated with severe acute respiratory syndrome. N. Engl. J. Med..

[3] Saad M., Omrani A.S., Baig K., Bahloul A., Elzein F., Matin M.A., A Selim M.A., Al Mutairi M., Al Nakhli D., Al Aidaroos A.Y. (2014). Clinical aspects and outcomes of 70 patients with Middle East respiratory syndrome coronavirus infection: A single-center experience in Saudi Arabia. Int. J. Infect. Dis..

[4] Ellul M.A., Benjamin L., Singh B., Lant S., Michael B.D., Easton A., Kneen R., Defres S., Sejvar J., Solomon T. (2020). Neurological associations of COVID-19. Lancet Neurol..

[5] Lauer S.A., Grantz K.H., Bi Q., Jones F.K., Zheng Q., Meredith H.R., Azman A.S., Reich N.G., Lessler J. (2020). The incubation period of coronavirus disease 2019 (COVID-19) from publicly reported confirmed cases: Estimation and application. Ann. Intern. Med..

[6] Wang D., Hu B., Hu C., Zhu F., Liu X., Zhang J., Wang B., Xiang H., Cheng Z., Xiong Y. (2020). Clinical characteristics of 138 hospitalized patients with 2019 novel coronavirus–infected pneumonia in Wuhan, China. JAMA.

[7] Huang C., Wang Y., Li X., Ren L., Zhao J., Hu Y., Zhang L., Fan G., Xu J., Gu X. (2020). Clinical features of patients infected with 2019 novel coronavirus in Wuhan, China. Lancet.

[8] Zhou F., Yu T., Du R., Fan G., Liu Y., Liu Z., Xiang J., Wang Y., Song B., Gu X. (2020). Clinical course and risk factors for mortality of adult inpatients with COVID-19 in Wuhan, China: A retrospective cohort study. Lancet.

[9] Cai Q., Chen F., Wang T., Luo F., Liu X., Wu Q., He Q., Wang Z., Liu Y., Liu L. (2020). Obesity and COVID-19 severity in a designated hospital in Shenzhen, China. Diabetes Care.

[10] Ceriello A., Stoian A.P., Rizzo M. (2020). COVID-19 and diabetes management: What should be considered?. Diabetes Res. Clin. Pract..

[11] Stoian A.P., Banerjee Y., Rizvi A.A., Rizzo M. (2020). Diabetes and the COVID-19 Pandemic: How Insights from Recent Experience Might Guide Future Management. Metab. Syndr. Relat. Disord..

[12] Wiersinga W.J., Rhodes A., Cheng A.C., Peacock S.J., Prescott H.C. (2020). Pathophysiology, Transmission, Diagnosis, and Treatment of Coronavirus Disease 2019 (COVID-19): A Review. JAMA.

[13] Hui L., Liang L., Dingyu Z., Jiuyang X., Huaping D., Nan T., Xiao S., Bin C. (2020). SARS-CoV-2 and viral sepsis: Observations and hypotheses. Lancet.

[14] Pinzon R.T., Wijaya V.O., Buana R.B., Al Jody A., Nunsio P.N. (2020). Neurologic Characteristics in Coronavirus Disease 2019 (COVID-19): A Systematic Review and Meta-Analysis. Front. Neurol..

[15] Mao L., Jim H., Wang M., Hu Y., Chen S., He Q., Chang J., Hong C., Zhou Y., Wang D. (2020). Neurologic Manifestations of Hospitalized Patients With Coronavirus Disease 2019 in Wuhan, China. JAMA Neurol..

[16] Helms J., Kremer S., Merdji H., Clere-Jehl R., Schenck M., Kummerlen C., Collange O., Boulay C., Fafi-Kremer S., Ohana M. (2020). Neurologic features in severe SARS-CoV-2 infection. N. Engl. J. Med..

[17] Nuzzo D., Picone P. (2020). Potential neurological effects of severe COVID-19 infection. Neurosci. Res..

[18] Moriguchi T., Harii N., Goto J., Harada D., Sugawara H., Takamino J., Ueno M., Sakata H., Kondo K., Myose N. (2020). A first case of meningitis/encephalitis associated with SARS-Coronavirus-2. Int. J. Infect. Dis..

[19] Carfì A., Bernabei R., Landi F. (2020). Persistent Symptoms in Patients After Acute COVID-19. JAMA.

[20] Carvalho-Schneider C., Laurent E., Lemaignen A., Beaufils E., Bourbao-Tournois C., Laribi S., Flament T., Ferreira-Maldent N., Bruyère F., Stefic K. (2021). Follow-up of adults with noncritical COVID-19 two months after symptom onset. Clin. Microbiol. Infect..

[21] Garrigues E., Janvier P., Kherabi Y., Le Bot A., Hamon A., Gouze H., Doucet L., Berkani S., Oliosi E., Mallart E. (2020). Post-discharge persistent symptoms and health-related quality of life after hospitalization for COVID-19. J. Infect..

[22] Taquet M., Luciano S., Geddes J.R., Harrison P.J. (2021). Bidirectional associations between COVID-19 and psychiatric disorder: Retrospective cohort studies of 62 354 COVID-19 cases in the USA. Lancet Psychiatry.

[23] Li Z., Liu T., Yang N., Han D., Mi X., Li Y., Liu K., Vuylsteke A., Xiang H., Guo X. (2020). Neurological manifestations of patients with COVID-19: Potential routes of SARS-CoV-2 neuroinvasion from the periphery to the brain. Front. Med..

[24] Li K., Wohlford-Lenane C., Perlman S., Zhao J., Jewell A.K., Reznikov L.R., Gibson-Corley K.N., Meyerholz D.K., McCray P.B. (2016). Middle east respiratory syndrome coronavirus causes multiple organ damage and lethal disease in mice transgenic for human dipeptidyl peptidase 4. J. Infect. Dis..

[25] Lochhead J.J., Kellohen K.L., Ronaldson P.T., Davis T.P. (2019). Distribution of insulin in trigeminal nerve and brain after intranasal administration. Sci. Rep..

[26] Song E., Zhang C., Israelow B., Lu-Culligan A., Prado A.V., Skriabine S., Lu P., Weizman O.-E., Liu F., Dai Y. (2021). Neuroinvasion of SARS-CoV-2 in human and mouse brain. J. Exp. Med..

[27] Fotuhi M., Mian A., Meysami S., Raji C.A. (2020). Neurobiology of COVID-19. J. Alzheimer’s Dis..

[28] Magrone T., Magrone M., Jirillo E. (2020). Focus on receptors for coronaviruses with special reference to angiotensin-converting enzyme 2 as a potential drug target—A perspective. Endocr. Metab. Immune Disord. Drug Targets.

[29] Karpiński T.M., Ożarowski M., Seremak-Mrozikiewicz A., Wolski H., Wlodkowic D. (2021). The 2020 race towards SARS-CoV-2 specific vaccines. Theranostics.

[30] Rhea E.M., Logsdon A.F., Hansen K.M., Williams L.M., Reed M.J., Baumann K.K., Holden S.J., Raber J., Banks W.A., Erickson M.A. (2021). The S1 protein of SARS-CoV-2 crosses the blood-brain barrier in mice. Nat. Neurosci..

[31] Lippi A., Domingues R., Setz C., Outeiro T.F., Krisko A. (2020). SARS-CoV -2: At the crossroad between aging and neurodegeneration. Mov. Disord..

[32] Di Carlo M., Giacomazza D., Picone P., Nuzzo D., San Biagio P.L. (2012). Are oxidative stress and mitochondrial dysfunction the key players in the neurodegenerative diseases?. Free Radic. Res..

[33] Picone P., Nuzzo D., Caruana L., Scafidi V., Di Carlo M. (2014). Mitochondrial dysfunction: Different routes to Alzheimer’s disease therapy. Oxid. Med. Cell. Longev..

[34] Nuzzo D., Picone P., Caruana L., Vasto S., Barera A., Caruso C., Di Carlo M. (2013). Inflammatory mediators as biomarkers in brain disorders. Inflammation.

[35] Nath A. (2020). Neurologic complications of coronavirus infections. Neurology.

[36] Merad M., Martin J.C. (2020). Pathological inflammation in patients with COVID-19: A key role for monocytes and macrophages. Nat. Rev. Immunol..

[37] Ahmadpoor P., Rostaing L. (2020). Why the immune system fails to mount an adaptive immune response to a COVID-19 infection. Transpl. Int..

[38] Li Y., Fu L., Gonzales D.M., Lavi E. (2004). Coronavirus neurovirulence correlates with the ability of the virus to induce proinflammatory cytokine signals from astrocytes and microglia. J. Virol..

[39] Koralnik I.J., Tyler K.L. (2020). COVID -19: A Global Threat to the Nervous System. Ann. Neurol..

[40] Connors J.M., Levy J.H. (2020). Thromboinflammation and the hypercoagulability of COVID-19. J. Thromb. Haemost..

[41] Mohamud A.Y., Griffith B., Rehman M., Miller D., Chelb A., Chebl A., Patel S.C., Howell B., Kole M., Marin H. (2020). Intraluminal carotid artery thrombus in COVID-19: Another danger of cytokine storm?. Am. J. Neuroradiol..

[42] Wijeratne T., Gillard-Crewther S., Sales C., Karimi L. (2021). COVID-19 pathophysiology predicts that ischemic stroke occurrence is an expectation, not an exception—A systematic review. Frontiers in Neurology. Front. Neurol..

[43] Al-Dalahmah O., Thakur K.T., Nordvig A.S., Prust M.L., Roth W., Lignelli A., Uhlemann A.-C., Miller E.H., Kunnath-Velayudhan S., del Portillo A. (2020). Neuronophagia and microglial nodules in a SARS-CoV-2 patient with cerebellar hemorrhage. Acta. Neuropathol. Commun..

[44] Sadeghi A., Tahmasebi S., Mahmood A., Kuznetsova M., Valizadeh H., Taghizadieh A., Nazemiyeh M., Aghebati-Maleki L., Jadidi-Niaragh F., Abbaspour-Aghdam S. (2021). Th17 and Treg cells function in SARS-CoV2 patients compared with healthy controls. J. Cell. Physiol..

[45] Lechien J.R., Chiesa-Estomba C.M., De Siati D.R., Horoi M., Le Bon S.D., Rodriguez A., Dequanter D., Blecic S., El Afia F., Distinguin L. (2020). Olfactory and gustatory dysfunctions as a clinical presentation of mild-to-moderate forms of the coronavirus disease (COVID-19): A multicenter European study. Eur. Arch. Oto Rhino Laryngol..

[46] Bénézit F., Le Turnier P., Declerck C., Paillé C., Revest M., Dubée V., Tattevin P., Arvieux C., Baldeyrou M., Chapplain J.-M. (2020). Utility of hyposmia and hypogeusia for the diagnosis of COVID-19. Lancet Infect. Dis..

[47] Varatharaj A., Thomas N., A Ellul M., Davies N.W.S., A Pollak T., Tenorio E.L., Sultan M., Easton A., Breen G., Zandi M. (2020). UK-wide surveillance of neurological and neuropsychiatric complications of COVID-19: The first 153 patients. Lancet Psychiatry.

[48] Lindan C.E., Mankad K., Ram D., Kociolek L.K., Silvera V.M., Boddaert N., Stivaros S.M., Palasis S. (2021). Neuroimaging manifestations in children with SARS-CoV-2 infection: A multinational, multicentre collaborative study. Lancet Child Adolesc. Health.

[49] Rogers J.P., Chesney E., Oliver D., A Pollak T., McGuire P., Fusar-Poli P., Zandi M.S., Lewis G., David A.S. (2020). Psychiatric and neuropsychiatric presentations associated with severe coronavirus infections: A systematic review and meta-analysis with comparison to the COVID-19 pandemic. Lancet Psychiatry.

[50] Berlit P., Bösel J., Gahn G., Isenmann S., Meuth S.G., Nolte C.H., Pawlitzki M., Rosenow F., Schoser B., Thomalla G. (2020). Neurological manifestations of COVID-19"-guideline of the German society of neurology. Neurol. Res. Pract..

[51] Phua J., Weng L., Ling L., Egi M., Lim C.-M., Divatia J.V., Shrestha B.R., Arabi Y.M., Ng J., Gomersall C.D. (2020). Intensive care management of coronavirus disease 2019 (COVID-19): Challenges and recommendations. Lancet Respir. Med..

[52] Portolés J., Marques M., López-Sánchez P., de Valdenebro M., Muñez E., Serrano M.L., Malo R., García E., Cuervas V. (2020). Chronic kidney disease and acute kidney injury in the COVID-19 Spanish outbreak. Nephrol. Dial. Transplant..

[53] Noris M., Benigni A., Remuzzi G. (2020). The case of complement activation in COVID-19 multiorgan impact. Kidney Int..

[54] Long B., Brady W.J., Koyfman A., Gottlieb M. (2020). Cardiovascular complications in COVID-19. Am. J. Emerg. Med..

[55] Disser N.P., De Micheli A.J., Schonk M.M., Konnaris M.A., Piacentini A.N., Edon D.L., Toresdahl B.G., Rodeo S.A., Casey E.K., Mendias C.L. (2020). Musculoskeletal Consequences of COVID-19. J. Bone Jt. Surg. Am..

[56] Sheehy L.M. (2020). Considerations for Postacute Rehabilitation for Survivors of COVID-19. JMIR Public Health Surveill..

[57] Liu K., Zhang W., Yang Y., Zhang J., Li Y., Chen Y. (2020). Respiratory rehabilitation in elderly patients with COVID-19: A randomized controlled study. Complement. Ther. Clin. Pract..

[58] Carda S., Invernizzi M., Bavikatte G., Bensmaïl D., Bianchi F., Deltombe T., Draulans N., Esquenazi A., Francisco G.E., Gross R. (2020). COVID-19 pandemic. What should Physical and Rehabilitation Medicine specialists do? A clinician’s perspective. Eur. J. Phys. Rehabil. Med..

[59] Nuzzo D., Cambula G., Bacile I., Rizzo M., Galia M., Mangiapane P., Picone P., Giacomazza D., Scalisi L. (2021). Long-Term Brain Disorders in Post Covid-19 Neurological Syndrome (PCNS) Patient. Brain Sci..

